# Knowledge and Awareness of Spanish Livestock Farmers of Antimicrobial Resistance: A Cross-Sectional Study

**DOI:** 10.3390/antibiotics15100949

**Published:** 2026-09-24

**Authors:** Sandra de Arozamena, Raquel Diez, Julen Susperregui, M. Jose Diez, Beatriz Romero, E. Milena Vazquez, Nelida Fernandez, Jose M. Rodriguez, Ana M. Sahagun

**Affiliations:** 1 Travelling Clinical Veterinary, 34100 Palencia, Spain; sdearo00@estudiantes.unileon.es; 2 Pharmacology, Department of Biomedical Sciences, Veterinary Faculty, Institute of Biomedicine (IBIOMED), University of Leon, 24071 Leon, Spain; rdielz@unileon.es (R.D.); mjdiel@unileon.es (M.J.D.); bromg@unileon.es (B.R.); evaza@unileon.es (E.M.V.); mnferm@unileon.es (N.F.); jmrodl@unileon.es (J.M.R.); 3 Applied Mathematics, Department of Mathematics, University of León, 24071 Leon, Spain; jsusl@unileon.es

**Keywords:** antibiotic use, antimicrobial resistance, awareness, behavior, farmer, food-producing animals, knowledge, livestock, Spain

## Abstract

Antimicrobial resistance is nowadays a global health issue, mainly related to antibiotic overuse and misuse in different sectors. In veterinary medicine, it is essential to understand farmers’ knowledge and awareness of this serious problem in order to minimize it. In Spain, there are virtually no studies on this issue. A cross-sectional online study was carried out among Spanish farmers to identify their knowledge and awareness of antibiotic use and antimicrobial resistance (AMR). Odds ratios (ORs) and 95% confidence intervals (95% CIs) were calculated, and logistic regression analysis was also performed. 217 farmers took part in the study, and 215 surveys were analyzed. Farmers were mainly men (60%), middle-aged (76.8%), and with more than 15 years of professional experience (41.4%). They worked mostly in extensive systems of production (53.5%), usually with only one species (54%), with ruminants being found in most farms (70.2%). Most participants showed a satisfactory knowledge of this topic, a positive attitude towards reducing the use of antibiotics, and a growing awareness of their importance for public health, although for almost a fifth of them the meaning of AMR is unclear. Respondents were more likely to achieve satisfactory scores if they were between 31 and 45 years (OR = 2.42, *p* < 0.05) and rearing only ruminant species (OR = 2.51, *p* < 0.05). Spanish livestock farmers have an acceptable level of knowledge about antibiotics and AMR, although there are still gaps related to the adequate use of these drugs.

## 1. Introduction

Antimicrobial resistance (AMR) is currently one of the main challenges for global public health in the 21st century [1]. Since the discovery of penicillin and the subsequent development of a large number of antibacterial agents during the so-called “golden age of antibiotic chemotherapy”, it has been possible to treat infections previously fatal in both humans and animals [2,3]. However, their widespread use in human medicine, animal health and agriculture has led to the problem we face nowadays. Although resistance mechanisms are natural evolutionary phenomena in bacteria, inappropriate practices such as use against non-bacterial infections, over- or under-dosing, the absence of accurate microbiological diagnosis, and non-compliance with health regulations, among others, have exacerbated them. All these factors have contributed to a progressive loss of efficacy of these drugs, so that mild infections may again become fatal [4].

In 2019, Murray et al. [5] pointed out that around 5 million deaths were related to AMR, with 1.3 million attributable to multidrug-resistant bacteria. It is also estimated that 10 million annual deaths will be reached by 2050 [6,7,8]. The European Centre for Disease Prevention and Control (ECDC) calculated that mortality would achieve 35,000 people/year by 2022 in the European Economic Area (EU/EEA), with an annual cost of up to €11.7 billion for healthcare systems [9,10]. In this context, the “One Health” approach is being promoted to address this problem as a whole, encompassing animal, human and environmental health [11]. In Spain, the National Plan against Antibiotic Resistance (PRAN) was launched in 2014 and is coordinated by the Spanish Agency for Medicines and Medical Devices (AEMPS) and the Ministry of Agriculture, Fisheries and Food (MAPA). PRAN, together with the corresponding European and international systems, establishes several lines of work based on communication, control and prevention, surveillance, research and training [12].

Livestock farming is a key sector in the food chain and is indispensable to control AMR [13]. In most Western countries, livestock farmers administer the majority of antimicrobial treatments after diagnosis and prescription by a veterinarian. In Spain, about 144,000 livestock farms were registered in 2023 [14], and around 800,000 people are carrying out their work there. As explained before, the responsible use of antimicrobials in this economic sector, as well as their proper disposal, are vital to prevent the spread of resistant microorganisms through food consumption and environmental contamination [13]. It should be borne in mind that the vast majority of antimicrobial drugs intended for livestock also have therapeutic value for human beings, and that resistant farm-acquired microorganisms can spread between humans and animals. On the other hand, AMR has a negative impact on livestock productivity, which can hinder safe food production [15].

Although veterinarians play a key role in reducing AMR, it has been shown in human medicine that intervention initiatives are aimed solely to improve knowledge and attitudes of prescribers often fail to address all the factors influencing the use of antimicrobials and may not have a lasting impact [16,17]. In this sense, active involvement of farmers in AMR is essential for reducing its prevalence. McKernan et al. (2021) [18] pointed out that prudent use of antimicrobials is associated with a better farmers’ knowledge of these drugs. In the case of Spain, and after a thorough review of the scientific literature, we have found only one study that evaluated in 2014 the opinions and attitudes of Spanish pig farmers regarding AMR [19]. However, in other countries more information is available, mainly focused on dairy farms [20,21,22,23,24], meat production [25], the control of specific diseases [24], or providing an overview of the current situation [26]. We believe it is essential to understand the habits, beliefs and knowledge of Spanish livestock farmers regarding the use of antimicrobials and, more specifically, antibiotics (these are the main group of antimicrobials used on livestock farms) as well as AMR. This information may help to understand those factors driving the use of antimicrobials in practice in order to ensure the responsible use of these drugs and help to minimize AMR on farms. For this reason, the present study aims to further assess the knowledge and awareness of Spanish livestock farmers towards AMR and their daily use of antibiotics.

## 2. Results and Discussion

A total of 217 farmers participated in the study, although 215 valid responses were obtained (two incomplete responses were removed from the final data set), exceeding the required sample size. Cronbach’s α was 0.716, which indicates an acceptable reliability and internal consistency of the questionnaire.

Most questionnaires were answered in 2024 (146 answers; 67.9%). Sociodemographic characteristics of respondents are shown in Table 1. Regarding gender, a slight predominance of men (60%) has been found, which is consistent with data reported in the 2023 Spanish Survey on the Structure of Agricultural Holdings by the Spanish Institute of Statistics (INE) [14] (71.3% male farm managers), highlighting the traditional masculinization of the livestock sector. As for age, most of them were middle-aged, mainly 18–30 (36.3%) and 31–45 years (40.5%). According to the INE [14], the majority of farm managers registered in Spain (86.4%) are over 44 years old. This discrepancy with our study may be linked to the fact that young people are more likely to use social media. As for gender and age, frequency among women was similar across all age groups of participants, with no significant differences observed between them (χ^2^ = 0.014; *p* = 0.993). When asked about the years of work experience as a farmer, 41.4% of participants have worked in farms for more than 15 years, whereas the groups with less than 5 years (24.2%) and between 5 and 10 years (22.3%) showed similar proportions. Again, no significant differences were revealed when gender and years of experience were compared (χ^2^ = 2.208; *p* = 0.530), nor between the production system used on the farms and participants’ gender (χ^2^ = 2.746; *p* = 0.253) or their age (χ^2^ = 7.460; *p* = 0.113).

The geographical distribution of farms is shown in Figure 1. Most of the answers came from the regions of Castilla y Leon (35.8%) and Galicia (15.8%), probably due to publicity generated by the questionnaire itself in the area of Leon, where it was launched. In 2023, more than half of all livestock farms were located in Galicia (19.1%), Andalusia (12.8%), Castilla and Leon (12.7%), and Extremadura (12.1%). Values are fairly similar when only farms specialized in ruminants are taken into account (Galicia 18.0%, Andalusia 13.4%, Castilla and Leon 13.2%, and Extremadura 12.2%) [14]. It should be noted that 4.2% of the participants selected several geographical regions, probably as they moved their livestock through them.

Table 2 details the characteristics of the farms. Extensive farming was the predominant self-perceived production system (53.5%), whilst semi-extensive and intensive farms each accounted for 23.3%. In Spain, most farms operate on a semi-extensive basis [27]. This hybrid approach allows for the efficient use of land to be combined with greater control over production and animal health. Regarding the productive animal species, a little over half of the farmers reported working with only one species (54%) and a quarter (25.1%) with three or more species. Ruminants were present in most farms (70.2%), clearly above non-ruminant (6.0%) or mixed farms (with both ruminant and non-ruminant animals, 23.7%). These data are coherent with the Spanish distribution of farms by type of livestock in 2023, with cattle in 79,278 (54.7%) of 144,939 registered farms, whereas sheep and goats were present in 51,080 (35.2%) and 24,096 farms (16.6%), respectively [14]. On the other hand, only three farms (1.4%) focused exclusively on poultry in our study, and five more (2.3%) raised poultry together with other non-poultry animals (cattle, sheep, goats, and pigs).

The results of the first two questions related to farmers’ knowledge of antibiotics and AMR consequences are summarized in Table 3. In general, an appropriate level of knowledge was observed, with most farmers correctly answering the definition of antibiotic (72.6%) and the annual deaths in Europe attributable to AMR (62.8%).

As for if they understood the concept of “antibiotic resistance” (open-ended question) (Figure 2), most of them associated this term with the lack or loss of efficacy against microorganisms (81.4%). However, it should be noted that almost a fifth are unsure what “antibiotic resistance” means, describing it as an animal’s body’s resistance to responding to these drugs (3.7%), the development of bacterial immunity against antibiotics (3.3%), the adaptation of bacteria to antibiotics or the strength of bacteria against these drugs (2.3% each), the animal’s failure to produce antibodies (0.9%) or linking it to incompatibility with the drugs (0.5%). It is also worrying that 5.1% was not able to answer the question despite their connection to the issue.

Some of these answers were provided regarding this term:

*The body either rejects the antibiotics or produces antibodies against them.*

*Bacteria living in the animal’s body learn to survive and develop an immune system against the antibiotics we administer; this can be caused by overuse of antibiotics.*

*The strength of the disease or bacteria to antibiotics.*

*Pathogens become resistant to the medicine, and it is necessary to make changes to it so that it can affect the animals’ ‘immune system’.*

It is also a cause of concern that some of the answers linked the concept of antibiotic resistance to the action of antibiotics against viruses (1.9%). Some answers in this regard were *The overuse or misuse of antibiotics causes the virus to become stronger and the body to build up a resistance to them, so they no longer work*, or *viruses last longer and antibiotics do not have the desired effect*.

On the other hand, distribution of answers to the 12 Likert-scale statements related to knowledge about antimicrobials and AMR are summarized in Figure 3. For these questions, a 4-point Likert scale was used as removing the midpoint from the scale prevents respondents from defaulting to an undecided position and encourages them to indicate whether their opinion leans towards the positive or the negative [28]. It should be highlighted that farmers clearly knew that antibiotics cannot be used for any animal disease (statement *they can be used for any animal disease*: strongly disagree 60.5%; 95% CI: 0.539–0.670; disagree 32.1%; 95% CI: 0.259–0.383), and that these compounds can be harmful to beneficial bacteria in the body (statement *they are not harmful to the beneficial bacteria in the body*: strongly disagree 45.6%; 95% CI: 0.389–0.522; disagree 45.1%; 95% CI: 0.385–0.518). They were also aware that antibiotic use can be reduced through good management practices and vaccination (strongly agree 52.1%; 95% CI: 0.454–0.588; agree 31.6%; 95% CI: 0.254–0.378), and that AMR detected in animals is relevant to public health (statement *antibiotic resistance in animals is not significant for public health*: strongly disagree 60.0%; 95% CI: 0.535–0.665; disagree 24.7%; 95% CI: 0.189–0.304).

In contrast, wrong responses were more frequent for other statements, which would suggest that certain misconceptions persist among farmers. In this regard, more than 20% thought that antibiotics do not eliminate bacteria (95% CI: 0.176–0.289), or can be effective against parasites, viruses or fungi (95% CI: 0.185–0.299). Moreover, more than a fifth still believed that the use of antibiotics on their farms would not affect them or their relatives (95% CI: 0.206–0.324), or they considered that AMR did not cause significant economic losses in livestock farming (95% CI: 0.189–0.304). What is even more worrying is that more than a quarter of farmers agreed that antibiotics relieve fever and pain (95% CI: 0.267–0.393), that frequent/inappropriate use of these drugs does not cause the emergence of AMR (95% CI: 0.272–0.398), or that any administration of antibiotics does not contribute to AMR (95% CI: 0.303–0.432). Moreover, more than half believed that restricting antibiotics in animals may do more harm than good (95% CI: 0.464–0.597). Nevertheless, most of them were aware that antibiotic treatment should not be discontinued if the animals’ health has improved (statement *once verified that the animal’s health has improved, I discontinue the treatment*: strongly disagree 46.5%; 95% CI: 0.398–0.532; disagree 35.8%; 95% CI: 0.294–0.422).

As for their attitude towards antibiotic use, assessed with the two final Likert-scale questions, when asked about the importance of reducing antibiotic use, they showed a strong predisposition towards prudent antimicrobial use practices: a large majority agreed that they were trying to reduce the use of antibiotics in their farms (strongly agree 54.0%; 95% CI: 0.473–0.606; agree 40.0%; 95% CI: 0.335–0.465), and they also believed that reducing antimicrobial use in livestock was important for human health (strongly agree 47.0%; 95% CI: 0.403–0.536; agree 44.7%; 95% CI: 0.380–0.513).

Taken together, these results reflect a predominantly positive attitude within farmers towards reducing the use of antibiotics, as well as a growing awareness of their importance for public health. The latest ESVAC (European Surveillance of Veterinary Antimicrobial Consumption) report [29] supports this approach, showing a 69.5% decrease in the use of antibiotics in Spanish livestock farming between 2014 and 2022. As for the recently introduced European initiative ESUAvet (European Sales and Use of Antimicrobials for Veterinary Medicine) [30], it does not yet allow for the analysis of more recent data. Limiting the use of antimicrobials is a key measure in curbing the development of AMR [31]. Nevertheless, bearing in mind that more than 50% believe that restricting the use of antibiotics in animals may be harmful, it appears that not all of them agree with the reduction achieved in antibiotic use. In Spain, antimicrobials must be prescribed by veterinarians. Moreover, since 2019, veterinarians have been required in this country to submit data related to antibiotic prescriptions for food-producing animals electronically to a national database [32]. Regulation (UE) 2019/6 [33] confirmed this requirement and extended it to pets, for which data has also been collected in this country since 2025 [34]. These and other measures, which form part of the Spanish PRAN program, may explain the reduction in antibiotic consumption achieved in this country over recent years.

Regarding scores achieved by participants, mean score was 20.8 ± 5.6 points (over 34). Scores by gender, age, years of experience as a farmer, type of livestock (ruminant/non-ruminant) and production system are shown in Figure 4. Values are higher in females, older farmers, those with 11–15 years of experience, and those specialized in ruminant animals and intensive farms. Moreover, as can be seen in Table 4, scores were clearly different between those achieving satisfactory and not satisfactory knowledge, with a higher proportion of livestock farmers who have satisfactory knowledge (76.7%). For all variables and groups considered, the frequency of farmers who achieved a satisfactory knowledge was higher than that of those with an unsatisfactory score, except in the case of the youngest participants (<18 years). However, this finding should be interpreted with caution, as only three participants of our study were included in this group. Scores achieved in both groups (satisfactory and not satisfactory) were higher in women, intensive farmers, and those specifically focused on ruminants. As for those respondents who gave an incorrect definition of resistance (13.0% of the total), almost a third (nine participants; 32.1%) scored low (<17 points). In this third with low scores, all of them were extensive farmers and mostly reared ruminants or both ruminants/non-ruminants.

Table 5 shows the results of the multivariate logistic regression analysis carried out. Only age and type of livestock had a significant impact on achieving satisfactory scores: being between 31 and 45 years made it 2.4 times more likely to score ≥ 17 points than younger farmers. As for the type of farm species, the probability to have adequate knowledge on the use of antibiotics (≥17 points) was 2.5 times significantly higher in farmers specialized in ruminant rearing than those whose farms reared all types of domestic animals. Older ages (>45 years) and rearing non-ruminant species were also related with satisfactory scores, but significant differences were not found.

In recent times, there has been growing interest in the knowledge and awareness of stakeholders involved in the use of antimicrobials. As explained before, farmers play a vital role in stemming AMR and must take part in reducing this threat. To do this, understanding farmers’ attitude towards recommended practices is necessary for reducing AMR [35]. Although over the last decade various papers have described the potential knowledge of farmers in different countries, a distinction should be drawn between high- and low-income countries. The lack of strong regulations in these latter countries means that farmers use self-prescription and get easily different antimicrobials. Furthermore, the absence of restrictions on their use as growth promoters or for prophylactic or metaphylactic purposes in many of these low-income developing countries, as well as the limited training received by livestock farmers in these countries, mean that it is not possible to compare the results obtained in most of these papers.

In the European Union, and also in Spain, considerable efforts have been made in recent decades to develop strategies aimed at preventing AMR and raising awareness of this issue. In the present study, just over three-quarters of the participants showed adequate knowledge about antibiotics and awareness of AMR, as described by other authors [36]. In contrast, Borelli et al. [20] estimated that only 50.1% of Scottish dairy farmers showed a satisfactory knowledge of antimicrobial usage and AMR. Other authors have also reported that farmers are aware that the increased use of antimicrobials contributed to the development of AMR [37,38,39]. Nevertheless, as in our study, ambivalence between the challenges posed by the day-to-day reality of their work and their responsibility regarding AMR has also been described [37].

The current study has shown that, as has happened in other European countries, there are still gaps or shortcomings in certain areas relating to the adequate use of antibiotics, such as the microorganisms that they actually target, the diseases or symptomatology for which they are effective, and the threat that AMR may pose to their economic activity or for the people around farmers [35,36,38,40,41,42]. These misconceptions that still remain may contribute to AMR development. In this regard, it is striking how many participants fail to recognize the link between the use of antibiotics in livestock and the potential consequences for their families. This highlights that, for some farmers, a lack of understanding of the AMR phenomenon may stem from the fact that being familiar with a particular subject or having knowledge of it does not necessarily mean that they understand it. It would therefore be necessary to emphasize practical guidance on the adequate use of these medicines in animals in order to tackle AMR, taking into account the specific characteristics of each farm (size, type of animals reared, production system, etc.). On the other hand, it seems that farmers have not perceived the threat that AMR caused by antimicrobial use in animals poses to human health. This skepticism about the contribution of livestock farming to AMR has been previously identified [37,43,44] and may pose a risk in terms of reducing AMR, as farmers will be less willing to devote time and resources to issues they do not perceive as a threat to their daily lives [45].

Demographic and production characteristics such as gender, age, and years of experience as a farmer tend to have a substantial impact on farmers’ knowledge and attitudes [46]. The degree of this relationship varies from one country to another [26]. In our study, age and the type of livestock were revealed to be important predictors of knowledge on antibiotics and AMR among Spanish farmers. Those of middle age or older or those specialized in the rearing of a certain type of livestock (ruminants/non-ruminants) show an adequate understanding of these drugs and AMR. Borelli et al. [20] pointed out that respondents breeding livestock other than dairy cows (beef and sheep) had better antimicrobial and AMR knowledge than dairy-only farmers, probably as they have been exposed to different awareness-raising campaigns and advisors.

The study is not without limitations, which should be taken into account when considering its applicability. On the one hand, the study is cross-sectional and captures information at a specific point in time; consequently, it does not allow us to establish a causal relationship or how farming practices might evolve over time. On the other hand, it was based on a self-administered questionnaire. So, caution should be taken when interpreting these results due to social desirability bias, as some responses may reflect what is perceived as the socially appropriate or desirable answer, rather than the participants’ actual practices. However, this bias may have been mitigated by the voluntary and anonymous nature of participation in the survey, which encourages people to answer honestly. The source of the questionnaire (social media) may also be considered a selection bias, as farmers who were more accessible, more willing to participate or more interested in the topic may have been over-represented, which would have led to an overestimation of their knowledge. It is also possible that some of the respondents were organic farmers, for whom the use of antibiotics is limited, and so perhaps some of them were already aware of the problem of AMR. Furthermore, it was carried out in a high-income country, which limits the generalizability of the results to a global level. Finally, farmers are one link in a complex network that includes consumers, veterinarians, traders and suppliers, among others, all of them involved in minimizing this threat.

## 3. Materials and Methods

### 3.1. Research and Questionnaire Design

An observational, descriptive, web-based cross-sectional study was conducted among Spanish livestock farmers. A Google form-based self-administered questionnaire was designed by the research team as an activity carried out within the project funded by the European Commission “Antimicrobial Resistance. A Threat and a European Priority (RESIST-UE)” (Jean Monnet Action Module, 101126992-RESIST-UE-ERASMUS-JMO-2023-HEl-TCH-RSCH). The questionnaire was designed after a comprehensive literature review to collect information on participant demographics, as well as their knowledge on antibiotics and AMR. The appropriateness of the questions raised was reviewed and discussed by all the authors. The questionnaire was written in Spanish and consisted of 12 questions grouped into three sections (Table S1).

The first section (6 questions) collected sociodemographic information from participants, including gender, age group, animal species reared, farm location, production system (intensive, extensive, semi-extensive), and years of farming experience. For the purpose of the study, productive species were classified as cattle, sheep/goats, horses, pigs, poultry, and others. The second section (3 questions) was focused on their knowledge of antibiotics and AMR. One of these three questions was an open-ended one, in which farmers had to explain what they understood as AMR. In the third section, knowledge, awareness and attitudes of farmers of antibiotics and AMR were requested through fifteen statements in three questions regarding the use of these drugs and the emergence of AMR, to be answered with a 4-point Likert scale (strongly agree, agree, disagree, or strongly disagree). The statements in question 10 were related to their knowledge on antibiotics and AMR, whereas the last 2 questions (11 and 12) were associated with their attitudes regarding this issue. A pilot study was previously conducted with 5 farmers to pretest it and verify that it was well understood. The time necessary to answer it was about 10 min. All participants received the same questionnaire, with the questions always shown in a fixed order. In all cases, questions referred to antibiotics rather than antimicrobials, as this term is more easily understood by farmers. The complete survey instrument can be found in the Supplementary Materials (Table S1).

The sample size was calculated based on the total number of livestock farms registered in Spain (144,939 in 2023 according to the Spanish Institute of Statistics) [14]. Assuming a precision of 0.07, an estimated probability of 0.5 and a significance level ≤ 0.05 [47], a minimum sample size of 196 livestock farmers was estimated. Data were reported according to the Strengthening the Reporting of Observational Studies in Epidemiology-Veterinary extension (STROBE-Vet) Statement [48].

### 3.2. Data Collection

As described before, the questionnaire was aimed at Spanish livestock farmers. The survey was available online between July 2024 and June 2025. The social network Instagram was used to promote the questionnaire, disseminate it and recruit as many participants as possible. A monthly monitoring of participation was made, and several reminders were sent through the same platform. Participation was voluntary and informed consent was requested before starting the survey. The questionnaire was also anonymous, the answers were coded to ensure confidentiality, and participants could stop filling in the survey whenever they wished. Data collection, processing, and storage were developed according to current Spanish regulations [49]. The study had been previously approved by the Ethics Committee of the University of León (ETICA-ULE-038-2024) and conducted in accordance with the principles established in the Declaration of Helsinki.

### 3.3. Data Analysis

Once obtained, data were processed and analyzed using Microsoft Excel 365 and SPSS Statistics 29 (IBM Corporation, Armonk, NY, USA). Internal consistency was assessed using Cronbach’s alpha [50]. Descriptive statistics (frequencies, mean, standard deviations, ranges, and percentages with 95% confidence intervals) were employed to characterize the study population. Chi-square (χ^2^) was used to compare qualitative variables (gender, group of age, years of experience as a farmer, and type of livestock). Multivariable forward-step logistic regression was also performed to identify those sociodemographic variables (gender, group of age, number of livestock species reared in the farm, ruminant/non ruminant species, production system, and years of farming activity) potentially associated with AMR. The odds ratio (OR) was calculated with their respective 95% confidence intervals (95% CIs). In all analyses, a statistical significance threshold of 5% was always used.

Answers were scored as explained below. In the second section of the questionnaire, the two multiple-choice questions were worth two points for each correct answer (0 points for all other options). Regarding the third section, as answers followed a 4 point-Likert-scale, scores were 2 (“totally” correct), 1 (partially correct) or 0 (wrong). Participants scoring 0–16 and 17–34 were graded as having “non-satisfactory” and “satisfactory” knowledge. The open-ended question of the second section, in which farmers explained what they understood as AMR, was not scored. Instead, answers were assessed independently by two of the authors to be coded. Any discrepancy was discussed by both researchers with a third author to reach a consensus.

## 4. Conclusions

In summary, the study shows that Spanish livestock farmers have an acceptable level of knowledge about antibiotics, although there is room for improvement in terms of both knowledge and awareness. Livestock farmers are, to a certain extent, aware of the AMR problem, although they do not fully understand the consequences this may have for themselves and those closest to them. Furthermore, they are trying to reduce their use of antibiotics, although they feel that this does not benefit them. This seems to leave scope for the authorities, livestock associations and veterinarians to improve training and dissemination strategies, so that farmers can overcome their preconceptions regarding the adequate use of these drugs, whilst ensuring their cooperation in disease prevention. The study also highlights the need for ongoing and comprehensive research into knowledge, perceptions and attitudes relating to the use of antibiotics to identify “at-risk” behaviors and the factors that may contribute to them, as potential targets for ongoing intervention aimed at optimizing and preserving the benefits of these drugs, whilst minimizing the risk to public health.

## Figures and Tables

**Figure 1:**
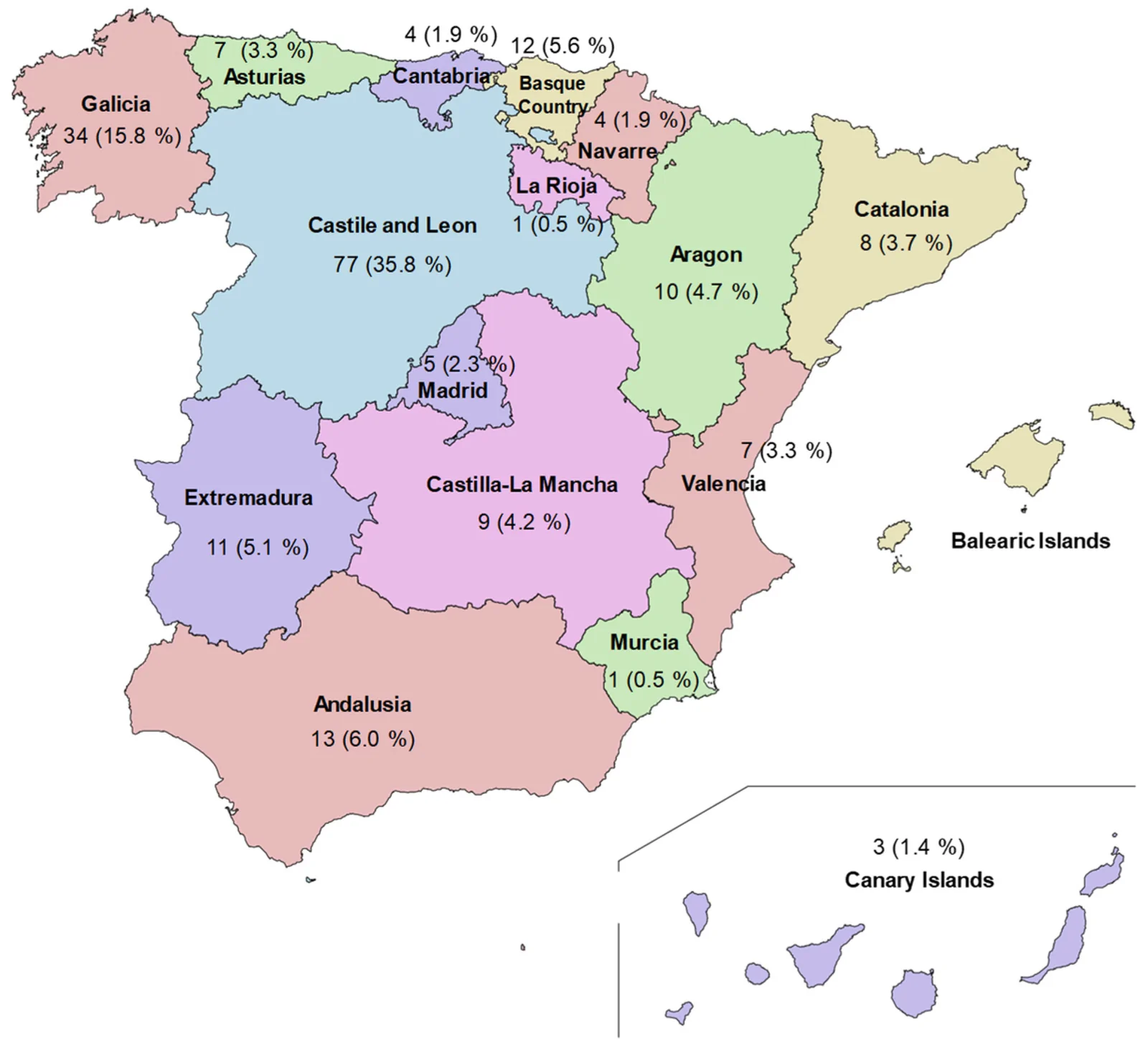
Geographical distribution of participants by Spanish regions (*n* and percentage).

**Figure 2:**
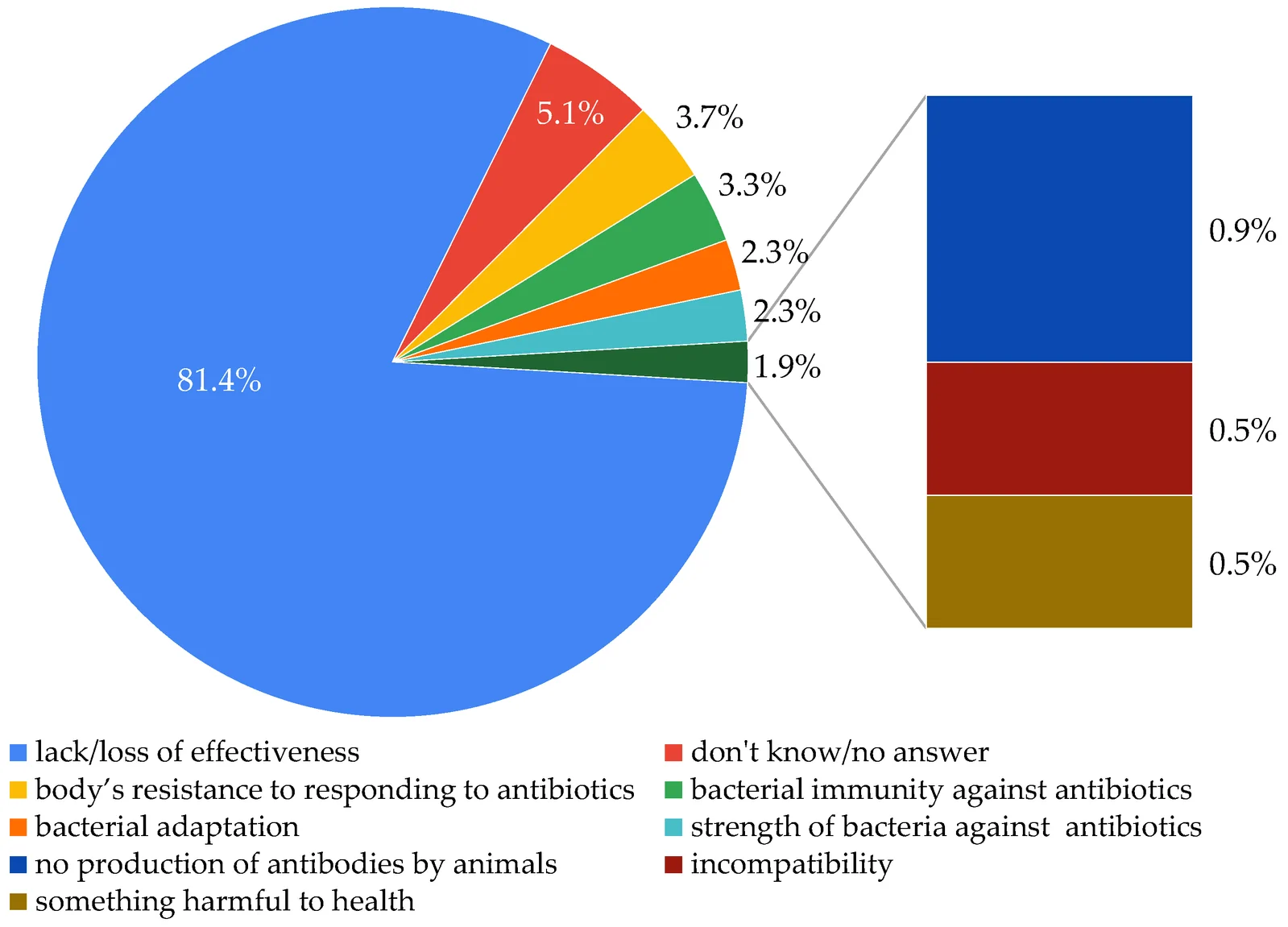
Answers provided by farmers on the concept of “antimicrobial resistance”.

**Figure 3:**
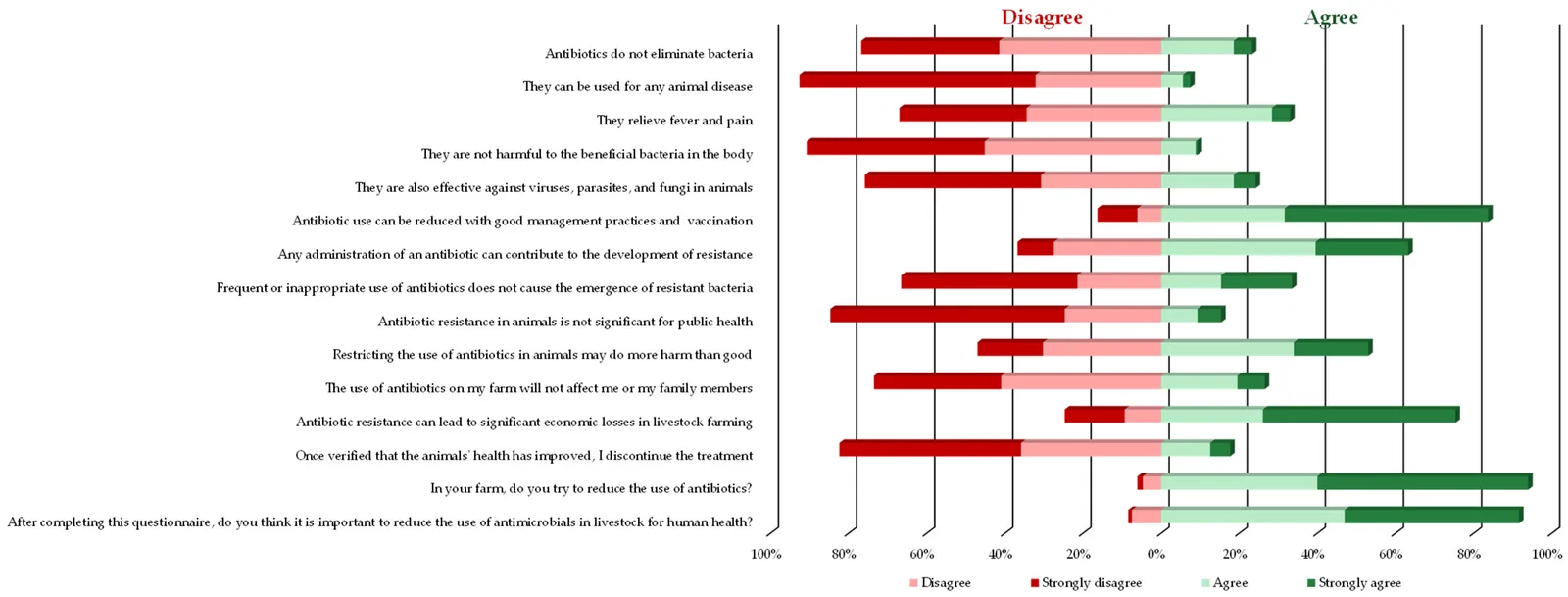
Answers provided by farmers on their knowledge and awareness of antibiotics.

**Figure 4:**
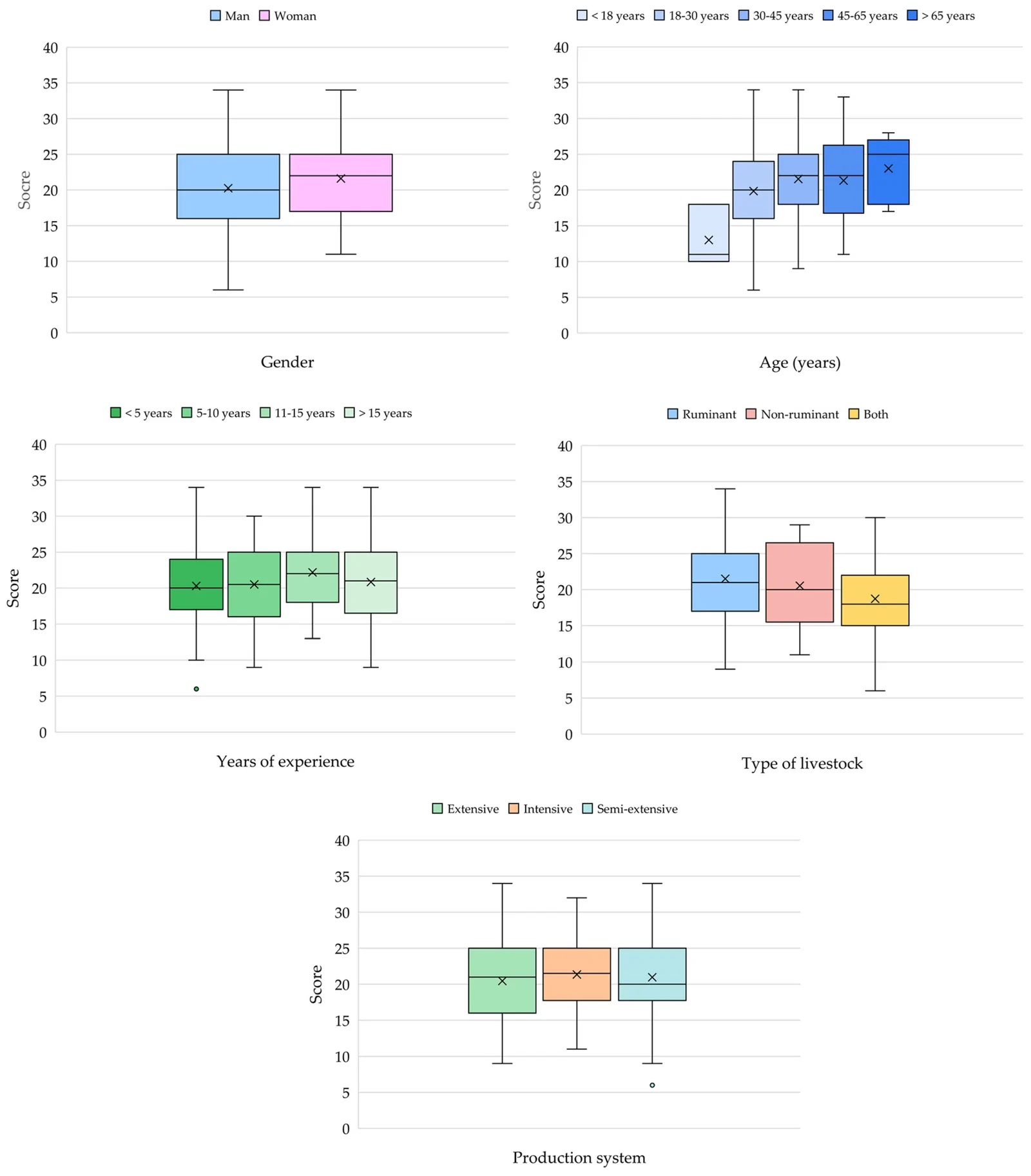
Scores achieved by farmers on antibiotic knowledge and AMR associated with demographic data and characteristics of the farms.

**Table 1:** Sociodemographic characteristics of the sample (Spanish farmers taking part in the study).

Characteristics	Frequency (%) (*n* = 215)	95% CI
Gender		
Male	129 (60.0)	0.535–0.665
Female	86 (40.0)	0.335–0.465
Age (years)		
<18	3 (1.4)	0.001–0.030
18–30	78 (36.3)	0.299–0.427
31–45	87 (40.5)	0.339–0.470
46–65	42 (19.5)	0.142–0.248
>65	5 (2.3)	0.003–0.043
Years of experience as a farmer		
<5	52 (24.2)	0.185–0.299
5–10	48 (22.3)	0.168–0.279
11–15	26 (12.1)	0.077–0.165
>15	89 (41.4)	0.348–0.480
Year of questionnaire answer		
2024	146 (67.9)	0.617–0.741
2025	69 (32.1)	0.298–0.380

CI: confidence interval.

**Table 2:** Characteristics of the farms belonging to participants.

Characteristics	Frequency (%) (*n* = 215)	95% CI
Production system		
Extensive	115 (53.5)	0.468–0.602
Intensive	50 (23.3)	0.176–0.289
Semi-extensive	50 (23.3)	0.179–0.289
No. of species reared		
1	116 (54.0)	0.473–0.606
2	45 (20.9)	0.155–0.264
≥3	54 (25.1)	0.193–0.309
Type of livestock		
Only ruminants	151 (70.2)	0.641–0.763
Only non-ruminants	13 (6.0)	0.029–0.092
Both	51 (23.7)	0.180–0.294

CI: confidence interval.

**Table 3:** Farmer knowledge of antibiotics and antimicrobial resistance (AMR).

Characteristics	Frequency (%) (*n* = 215)	95% CI
What do you mean by antibiotic?		
A drug to kill bacteria	36 (16.7)	0.118–0.217
A drug to prevent bacteria from multiplying	14 (6.5)	0.032–0.098
A drug to kill parasites, fungi, and viruses	9 (4.2)	0.015–0.069
The first two answers are correct	156 (72.6)	0.666–0.785
How many people die annually in Europe due to AMR?		
35,000	135 (62.8)	0.563–0.693
5000	58 (27.0)	0.210–0.329
1000	14 (6.5)	0.032–0.098
10	8 (3.7)	0.012–0.063

CI: confidence interval.

**Table 4:** Scores achieved in the outcome variables employed in the regression model assessing Spanish farmers’ knowledge of antimicrobial use and resistance.

Outcome Variable	Satisfactory ($\bar{x}$ ± SD) (%)	Non-Satisfactory ($\bar{x}$ ± SD) (%)
	23.0 ± 4.2 (76.7%)	13.5 ± 2.4 (23.3%)
Gender		
Men	22.8 ± 4.0 (74.4%)	12.8 ± 2.5 (25.6%)
Women	23.4 ± 4.4 (80.2%)	14.8 ± 1.5 (19.8%)
Age (years)		
<18	18.0 – (33.3%)	10.5 ± 0.7 (66.7%)
18–30	22.6 ± 4.0 (70.5%)	13.2 ± 2.9 (29.5%)
31–45	23.1 ± 4.1 (82.8%)	14.2 ± 1.9 (17.2%)
46–65	23.7 ± 4.5 (76.2%)	13.6 ± 1.4 (23.8%)
>65	23.0 ± 4.7 (100.0%)	-
Years of experience as a farmer		
<5	22.4 ± 4.0 (76.0%)	13.3 ± 3.0 (24.0%)
6–10	23.1 ± 4.2 (70.8%)	14.1 ± 2.5 (29.2%)
11–15	22.8 ± 4.4 (92.3%)	14.5 ± 2.1 (7.7%)
>15	23.4 ± 4.2 (75.3%)	13.0 ± 2.0 (24.7%)
Production system		
Extensive	23.0 ± 4.2 (73.9%)	13.3 ± 2.2 (26.1%)
Intensive	23.3 ± 3.8 (78.0%)	14.4 ± 2.1 (22.0%)
Semi-extensive	22.8 ± 4.5 (82.0%)	12.8 ± 3.3 (18.0%)
Type of livestock		
Only ruminants	23.5 ± 4.3 (80.1%)	13.6 ± 2.4 (19.9%)
Only non-ruminants	23.1 ± 4.4 (76.9%)	12.0 ± 1.0 (23.1%)
Both	21.4 ± 3.4 (66.7%)	13.5 ± 2.6 (33.3%)

Non-satisfactory scores: 0–16 points; satisfactory scores: 17–34 points. $\bar{x}$: mean; SD: standard deviation.

**Table 5:** Multivariate logistic regression analysis showing the association between predictor variables and the possibility of achieving a score of 17 or higher (satisfactory knowledge).

Variables		OR	95% CI	*p*
Age (years)	31–45	2.416	1.246–4.682	0.009 *
	>45	1.949	0.888–4.276	0.096
Type of livestock	Only ruminants	2.509	1.519–4.144	<0.001 *
	Only non-ruminants	2.036	0.525–7.888	0.304

* Significant differences (*p* ≤ 0.05). CI: confidence interval; OR: odds ratio.

## Data Availability

The data presented in this study are available within the article. No additional datasets were generated.

## Supplementary Materials

The following supporting information can be downloaded at: https://www.mdpi.com/article/10.3390/antibiotics15100949/s1, Table S1: Questionnaire used in this study.

## Abbreviations

The following abbreviations are used in this manuscript:

AEMPS	Spanish Agency for Medicines and Medical Devices (Agencia Española de Medicamentos y Productos Sanitarios)
AMR	Antimicrobial resistance
CI	Confidence Interval
ECDC	European Centre for Disease Prevention and Control
ESUAvet	European Sales and Use of Antimicrobials for Veterinary Medicine
ESVAC	European Surveillance of Veterinary Antimicrobial Consumption
EU/EEA	European Economic Area
INE	Spanish Institute of Statistics (Instituto Nacional de Estadística)
MAPA	Ministry of Agriculture, Fisheries and Food (Ministerio de Agricultura, Pesca y Alimentación)
PRAN	National Plan against Antibiotic Resistance (Plan Nacional Resistencia Antibióticos)
OR	Odds Ratio
STROBE-Vet	Strengthening the Reporting of Observational Studies in Epidemiology-Veterinary
