# Supplementary material for: Knowledge and Awareness of Spanish Livestock Farmers of Antimicrobial Resistance: A Cross-Sectional Study
Source: Antibiotics (Basel). 2026 Sep 24;15(10):949. doi: 10.3390/antibiotics15100949 (PMC13700766; doi:10.3390/antibiotics15100949)
Supplement: Supplementary file 1 [file antibiotics-15-00949-s001.zip › antibiotics-4571141-supplementary.pdf]

Table S1. Questionnaire used in this study.

QUESTIONNAIRE

Antibiotic Resistance: Knowledge and Attitudes amongst Livestock Farmers

1. Gender

☐ Woman ☐ Man ☐ Other: ______________________________

2. Age

☐ < 18 years ☐ 18-30 years ☐ 31-45 years

☐ 46-65 years ☐ < 65 years

3. Animal species reared

☐ Bovine ☐ Sheep/Goat ☐ Equine

☐ Poultry ☐ Swine

☐ Other: __________________________

4. Spanish province where your farm is located ______________________

5. Production system of your farm

☐ Intensive ☐Extensive ☐ Semi-extensive

6. Years of experience as a farmer

☐ >5 years ☐ 5-10 years ☐ 11-15 years ☐ < 15 years

7. What do you mean by "antibiotic"?

☐ A drug to kill bacteria

☐ A drug to prevent bacteria from multiplying

☐ A drug to kill parasites, fungi, and viruses

☐ The first two answers are correct

8. What do you understand by "antibiotic resistance"? ___________________--

9. How many people die annually in Europe due to AMR?

☐ 35,000 ☐ 10 ☐ 5,000 ☐ 1,000

10. For the following statements, please select the option that best reflects your opinion:

	Strongly disagree	Disagree	Agree	Strongly agree
Antibiotics do not eliminate bacteria	☐	☐	☐	☐
They can be used for any animal disease	☐	☐	☐	☐
They relieve fever and pain	☐	☐	☐	☐

	Strongly disagree	Disagree	Agree	Strongly agree
They are not harmful to the beneficial bacteria in the body	☐	☐	☐	☐
They are also effective against viruses, parasites, and fungi in animals	☐	☐	☐	☐
Antibiotic use can be reduced with good management practices and vaccination	☐	☐	☐	☐
Any administration of an antibiotic can contribute to the development of resistance	☐	☐	☐	☐
Frequent or inappropriate use of antibiotics does not cause the emergence of resistant bacteria	☐	☐	☐	☐
Antibiotic resistance in animals is not significant for public health	☐	☐	☐	☐
Restricting the use of antibiotics in animals may do more harm than good	☐	☐	☐	☐
The use of antibiotics on my farm will not affect me or my family members	☐	☐	☐	☐
Antibiotic resistance can lead to significant economic losses in livestock farming	☐	☐	☐	☐
Once verified that the animals' health has improved, I discontinue the treatment	☐	☐	☐	☐

11. In your farm, do you try to reduce the use of antibiotics?

☐ Strongly disagree ☐ Disagree

☐ Agree ☐ Strongly agree

12. After completing this questionnaire, do you think it is important to reduce the use of antimicrobials in livestock for human health?

☐ Strongly disagree ☐ Disagree

☐ Agree ☐ Strongly agree
